# Impact of vaginal microbiome communities on HIV antiretroviral-based pre-exposure prophylaxis (PrEP) drug metabolism

**DOI:** 10.1371/journal.ppat.1009024

**Published:** 2020-12-03

**Authors:** Ryan K. Cheu, Andrew T. Gustin, Christina Lee, Luca Schifanella, Charlene J. Miller, Avie Ha, Casey Kim, Violeta J. Rodriguez, Margaret Fischl, Adam D. Burgener, Kelly B. Arnold, Maria L. Alcaide, Nichole R. Klatt

**Affiliations:** 1 Department of Pharmaceutics, University of Washington, Seattle, Washington, United States of America; 2 Washington National Primate Research Center, University of Washington, Seattle, Washington, United States of America; 3 Department of Pediatrics, University of Miami Miller School of Medicine, Miami, Florida, United States of America; 4 Division of Surgical Outcomes and Precision Medicine Research, Department of Surgery, University of Minnesota, Minneapolis, Minnesota, United States of America; 5 Department of Biomedical Engineering, University of Michigan, Ann Arbor, Michigan, United States of America; 6 Department of Medicine, University of Miami Miller School of Medicine, Miami, Florida, United States of America; 7 Department of Psychology, University of Georgia, Athens, Georgia, United States of America; 8 Department of Medical Microbiology and Infectious Diseases, University of Manitoba, Winnipeg, Manitoba, Canada; 9 Center for Global Health and Disease, Case Western Reserve University, Cleveland, Ohio, United States of America; Vaccine Research Center, UNITED STATES

## Abstract

Despite the efficacy of antiretroviral-based pre-exposure prophylactics (PrEP) in men who have sex with men, studies in women have produced widely varying outcomes. Recent evidence demonstrates that vaginal microbial communities are associated with increased HIV acquisition risk and may impact PrEP efficacy. Here, we investigate the mechanisms underlying how vaginal bacteria alter PrEP drug levels and impact HIV infection rates *ex vivo*. Using cervicovaginal lavages (CVLs) from women with or without bacterial vaginosis (BV), we identified microbial metabolism of PrEP drugs in BV samples through LC-MS/MS analysis of soluble drug levels and metabolite formation in dual T-cell cultures. CVL samples were assessed for microbiome analysis using sequencing of bacterial 16S rRNA genes. We also observed non-*Lactobacillus* bacteria that are associated with BV may potentially impact PrEP efficacy through increased HIV infection rates in co-cultures containing *Lactobacillus* or BV bacteria, PrEP drugs, CEM-GFP cells, and HIV-1_LAI_ virus. Finally, we used these data to develop a novel predictive mathematical simulation modeling system to predict these drug interactions for future trials. These studies demonstrate how dysbiotic vaginal microbiota may impact PrEP drugs and provides evidence linking vaginal bacteria to PrEP efficacy in women.

## Author summary

HIV prevention strategies with antiretroviral drugs as pre-exposure prophylactics (PrEP) are not efficacious in women, in part due to biological factors such as the vaginal microbiome. *Lactobacillus* spp. prevent the colonization of dysbiotic bacteria (i.e. bacterial vaginosis), which is associated with increased HIV transmission. However, the role these microbes play in altering the pharmacokinetics of PrEP drugs is currently unknown. Here we show that primary bacteria from women with dysbiosis impact PrEP drug levels and kinetics. We found that dysbiotic microbes metabolize PrEP drugs faster than target cell uptake, and significantly alter HIV infection rates *in vitro*, and this can be predicted with novel computational modeling. Our results demonstrate that the vaginal microbiome may play a key role in HIV prevention through altering therapeutic drug levels via metabolism. Thus, better measurements and interventions for vaginal dysbiosis will be critical in improving the efficacy of HIV prevention efforts in women.

## Introduction

Women account for more than half of the 35 million people living with human immunodeficiency virus (HIV), and young women are among the most vulnerable to HIV acquisition worldwide. Women contribute to over 1 million new infections annually, with the majority of these new infections located in sub-Saharan Africa [1]. In 2016, close to 18 million women were living with HIV [2]. With no efficacious vaccine, HIV prevention strategies are essential for prevention of HIV transmission. Nevertheless, while oral PrEP with Tenofovir/Emtricitabine (TDF/FTC) has demonstrated efficacy in men who have sex with men (MSM) [3], both oral and topical PrEP have yielded highly variable efficacy results for women [4]. This variability has been attributed to adherence or the presence of semen, however other biological issues such as the vaginal microbiome can play a role. Recent studies have highlighted the role these microbes can play in diminishing the efficacy of HIV prevention for women [4].

In the female reproductive tract (FRT), *Lactobacillus* spp. prevent colonization by pathogenic bacteria and are associated with protection from HIV [5–7]. However, colonization of the FRT by more diverse communities of anaerobic bacteria, notably *Gardnerella vaginalis*, is common [8] and is associated with the development of bacterial vaginosis (BV) [8]. Recent studies have demonstrated that loss of *Lactobacillus spp*. replaced with high diversity bacteria in the FRT strongly correlates with increased pro-inflammatory cytokines as a potential mechanism by which cervicovaginal microbiota may increase the risk of HIV acquisition in women via vaginal intercourse [9]. BV is highly prevalent, affecting 4–58% of women globally, with rates as high as 55% in sub-Saharan Africa [5,10,11]. In fact, a study in Zambia demonstrated 63.3% of women had BV [12]. BV is associated with significant obstetric and gynecological poor health outcomes, and with increased susceptibility to STIs [5] and HIV [8,13]. BV is associated with a 60% increase in HIV acquisition rates [5,10,11] however the mechanisms that underlie increased acquisition, particularly in the context of PrEP use, are not clearly understood. In a study of young, healthy South African women, investigators found that *G*. *vaginalis*-containing high diversity anaerobic dysbiosis was associated with a 4-fold higher risk of acquiring HIV and had elevated genital CD4+ T cells compared with *Lactobacillus spp*. dominant women [6]. Of note, amongst *Lactobacillus* spp., *L*. *iners* has been shown to be more pro-inflammatory and, when compared with *L*. *crispatus* or *L*. *jensenii*, differ in their ability to produce hydrogen peroxide, Lactic acid isoforms, and in their role in protecting against STIs [14]. Maintenance of the mucosal barrier is critical for preventing invading microorganisms, including HIV, and bacterial diversity in the FRT has been strongly associated with FRT inflammation that negatively impacts FRT vulnerability to HIV infection [15]. Indeed, genital inflammation can undermine PrEP efficacy [16].

Another potential role of the vaginal microbiome on HIV acquisition in the context of PrEP may be via alteration of pharmacokinetics of PrEP drugs. Indeed, recent studies from us and others have highlighted the important role of drug metabolism by bacteria and the potential role microbiome-mediated metabolism may play in drug efficacy [4,17–19]. PrEP is a strategy aimed at preventing HIV infection by using antiretrovirals (ART) to prevent viral replication before infection. While PrEP has demonstrated to be highly effective in preventing the acquisition of HIV infection in men who have sex with men (MSM), studies in women have produced suboptimal outcomes. Studies evaluating topical PrEP with 1% tenofovir have shown low levels of protection (from 0% in FACTS to 39% in the Center for AIDS Program of Research in South Africa CAPRISA 004 trial) [20,21]. Daily oral PrEP (FTC/TDF) demonstrated results ranging from -49% reduction (VOICE) to 75% reduction (TDF2) [20]. Differences in outcomes between these prevention trials were attributed to adherence, but recent data suggest that other biological factors at the mucosal level and the composition of the vaginal microbiome contributed to the variability in PrEP efficacy as well [4,22]. A secondary analysis of the CAPRISA 004 trial demonstrated differences in efficacy based on vaginal microbial communities and identified taxa responsible for bacteria-mediated drug metabolism that likely contributed to efficacy [4].

Truvada is the first FDA drug available for PrEP use. This prophylaxis treatment is a combination of tenofovir disoproxil fumarate (TDF) and emtricitabine (FTC). TDF is the prodrug form of tenofovir (TFV), containing an additional ester group to improve drug absorption. Topical microbicides, like the TFV vaginal ring, are not in the prodrug formulation, and thus our study uses TFV in its original form. While we focused on this specific drug, our study also includes the use of additional PrEP drugs such as tenofovir alafenamide (TAF) and dapivirine (DPV). TAF is the next generation oral PrEP drug that is also a pro-drug of TFV. TAF contains an amide group that aims to achieve therapeutic levels through lower dosage forms due to increasing lipophilicity. DPV is also a topical microbicide being tested in clinical trials as a vaginal ring. Unlike TAF and TFV, DPV is a non-nucleotide reverse transcriptase inhibitor (NNRTI). Due to the absence of an adenine group, we were interested in studying DPV to test *G*. *vaginalis* drug-metabolism potential on this drug. DPV has been used in two major trials, ASPIRE and the Ring Study, where DPV vaginal rings were used for HIV prevention, with efficacy of 37% and 31%, respectively [23,24].

To improve the efficacy of prevention strategies for HIV in women, it is critical to define the mechanisms by which vaginal microbiome affects PrEP drug levels. In this study, we investigate how vaginal bacteria alter PrEP drug concentrations through drug metabolism. Based on our previous work with CAPRISA 004 trial [4], we hypothesize that drug metabolism by vaginal bacteria may contribute to the variability in PrEP efficacy. Specifically, we hypothesized that BV associated bacteria, such as *G*. *vaginalis*, decrease extracellular drug levels and prevent intracellular accumulation of active drug metabolites. We use an ordinary differential equation-based (ODE) model to 1) quantify internalization rates of TAF, TFV, and DPV for target cells and *G*. *vaginalis*; and 2) illustrate how drug metabolism changes relative to microbial community compositions over time. The data presented here indicate that variability seen in PrEP drug trials may not only be due to varying adherence, inflammation and other biological factors, but also to vaginal microbial composition and PrEP drug metabolism by dysbiotic bacteria.

## Results

Cervicovaginal lavage samples from forty-four women were analyzed using 16S ribosomal RNA (rRNA) sequencing, which identified 352 different bacteria genera. Fifteen women were diagnosed with BV based on Nugent score above 7, while twenty-nine were BV negative by Nugent score under 7. Thirty-three of these women were HIV positive, while eleven were HIV negative. Two major bacterial community groups were identified: one in which *Lactobacillus* dominated (>50%, n = 18 women) and the other dominated by non-*Lactobacillus* microbiota (<50%, n = 26 women) (S1 Table).

### Effect of vaginal dysbiosis on bacteria-mediated drug metabolism

**Fig 1:**
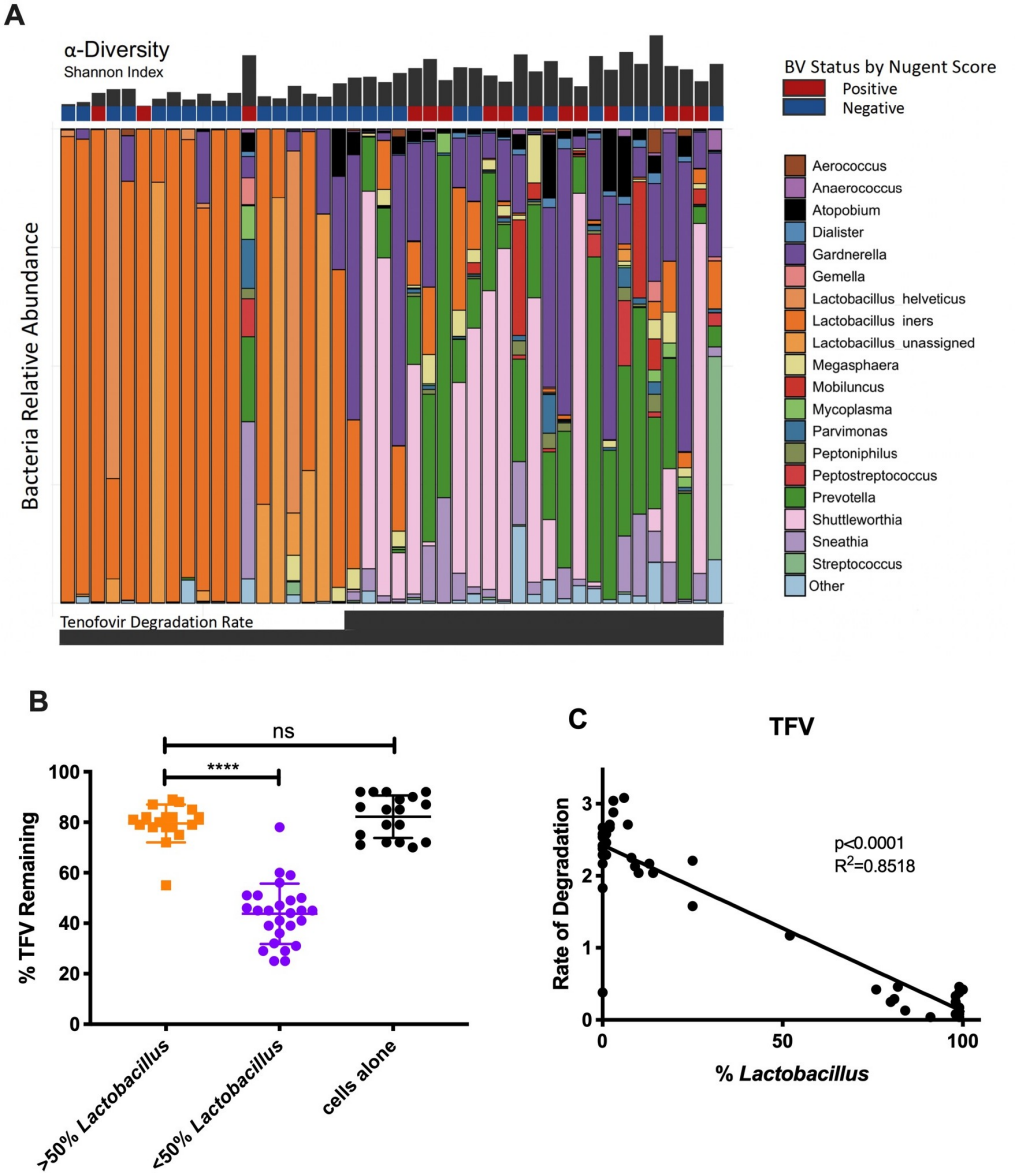
Effects of vaginal bacteria on TFV degradation. (A)Relative abundance of CVLs from 44 women with and without diagnosed BV ranked in order of increasing TFV degradation rates. The 19 most abundant phyla are shown. Shannon diversity plots showing alpha diversity in CVLs. Blue, BV negative by Nugent score at the time of collection; Red, BV positive by Nugent score at the time of collection. (B) % TFV remaining after 24 hours at 37C in *Lactobacillus* dominant vs. non-*Lactobacillus* dominant CVLs. Orange, greater than 50% dominance; Purple, less than 50% dominance; Black, cells + TFV control. (C) Rate of TFV degradation (% lost/hour) vs. %*Lactobacillus* in CVLs after 24-hour incubation. Rate of degradation calculated as % remaining/24 hours.

To identify the effects of vaginal bacteria on bacteria-mediated drug metabolism, CVL samples were incubated with Jurkat cells with three different ART-based PrEP drugs, and bacteria communities were determined by 16S rRNA sequencing. Since some participants were HIV positive taking ART drugs, all CVLs were tested for residual ART drug levels prior to analysis and no CVLs had detectable drug levels prior to drug metabolism assays. Bacteria were tested and confirmed for viability using selective media for *Lactobacillus* spp. and *G*. *vaginalis*. Soluble drug levels were measured after samples were subjugated to centrifugation and the supernatants were assessed. Fig 1 identifies the effects these microbes can have on TFV. Fig 1A ranks the samples in order from the least amount of TFV degraded and shows the bacterial community composition for each sample. To identify the effects of *Lactobacillus* on drug biodegradation, we looked at differences in % TFV remaining between *Lactobacillus* dominant versus non-*Lactobacillus* dominant. In samples with less than 50% *Lactobacillus*, we observed only 43.8±11.9% remaining of TFV (p<0.0001) after 24 hours when compared to 79.6±7.5% remaining for *Lactobacillus* dominant samples (Fig 1B). Our control group without bacteria from the CVLs demonstrated 82.2±8.4% remaining, which was not statistically significantly different from the *Lactobacillus* dominant samples (p>0.9999; Fig 1B). Our study also found that rates of degradation for TFV exhibited a significant negative correlation relative to *Lactobacillus* abundance (R^2^ = 0.8518, p<0.0001; Fig 1C).

**Fig 2:**
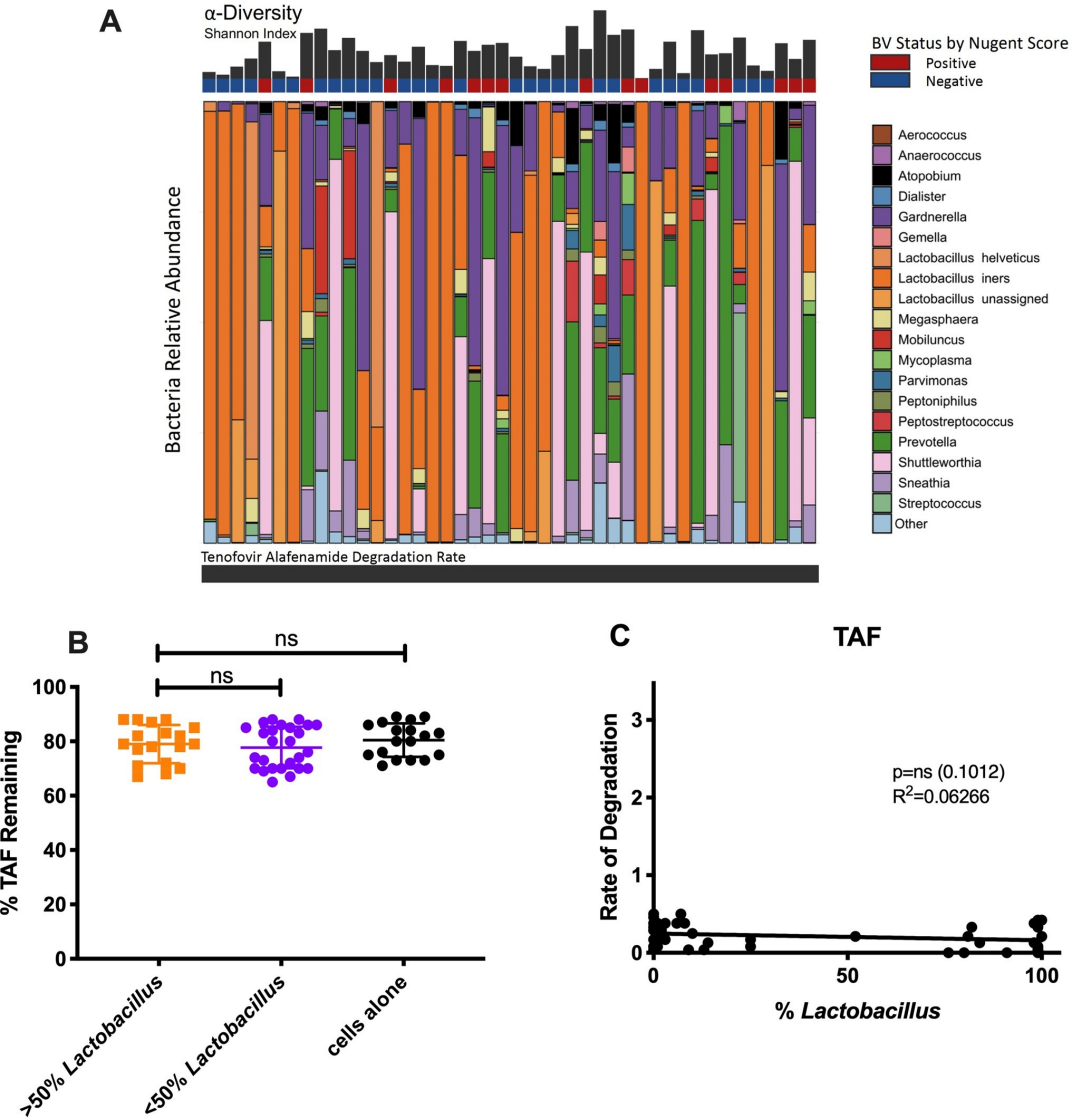
Effects of vaginal bacteria on TAF degradation. (A) Relative abundance of CVLs from 44 women with and without diagnosed BV ranked in order of increasing TAF degradation rates. The 19 most abundant phyla are shown. Shannon diversity plots showing alpha diversity in CVLs. Blue, BV negative by Nugent score at the time of collection; Red, BV positive by Nugent score at the time of collection. (B) % TAF remaining after 24 hours at 37C in *Lactobacillus* dominant vs. non-*Lactobacillus* dominant CVLs. Orange, greater than 50% dominance; Purple, less than 50% dominance; Black, cells + TFV control. (C) Rate of TAF degradation (% lost/hour) vs. %*Lactobacillus* in CVLs after 24-hour incubation. Rate of degradation calculated as % remaining/24 hours.

Next, we evaluated the effects of vaginal bacteria on the pro-drug tenofovir alafenamide (TAF), given that this is a next-generation drug being newly prescribed for PrEP. We found no significant difference in the degradation rate of TAF, regardless of bacteria community state (Fig 2). Similarly, we found no difference in % TAF remaining between our *Lactobacillus* dominant (79±7.0%) and non-*Lactobacillus* dominant samples (77.7±7.6%, p = 0.1156; Fig 2) as well as no association between rate of degradation and fraction of *Lactobacillus* (Fig 2). Our control group matched both experimental in %TAF remaining (80.4±6.2%).

**Fig 3:**
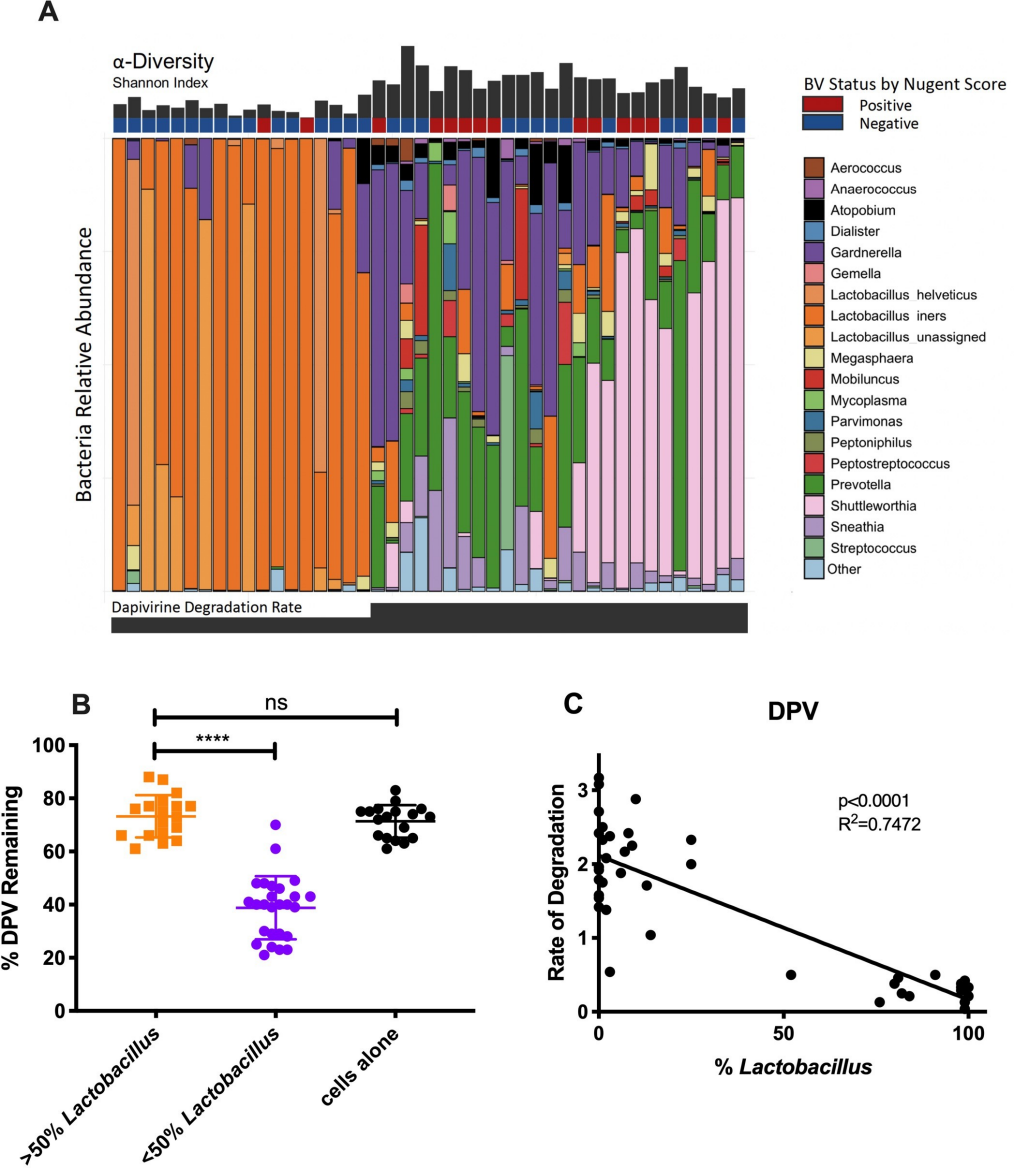
Effects of vaginal bacteria on DPV degradation. (A) Relative abundance of CVLs from 44 women with and without diagnosed BV ranked in order of increasing DPV degradation rates. The 19 most abundant phyla are shown. Shannon diversity plots showing alpha diversity in CVLs. Blue, BV negative by Nugent score at the time of collection; Red, BV positive by Nugent score at the time of collection. (B) % DPV remaining after 24 hours at 37C in *Lactobacillus* dominant vs. non-*Lactobacillus* dominant CVLs. Orange, greater than 50% dominance; Purple, less than 50% dominance; Black, cells + TFV control. (C) Rate of DPV degradation (% lost/hour) vs. %*Lactobacillus* in CVLs after 24-hour incubation. Rate of degradation calculated as % remaining/24 hours.

Dapivirine (DPV) has been used in trials of vaginal ring-based PrEP [25,26]. Fig 3A highlights the effects that dysbiotic bacteria have on DPV degradation, with a clear association between diversity of vaginal bacterial communities and increased DPV degradation. When categorizing samples between *Lactobacillus*-dominant versus non-*Lactobacillus* dominant, we identified a significant increase in DPV remaining after incubation in samples with *Lactobacillus* dominance (p<0.0001; Fig 3B). We saw an average of 73.2±7.96% DPV remaining in our *Lactobacillus*-dominant samples versus only 38.8±11.9% in our non-*Lactobacillus* dominant samples. Our control group without bacteria found 71.3±6.1% remaining after incubation. Our study also found that rates of degradation for DPV were also negatively associated with *Lactobacillus* abundance (R^2^ = 0.7274, p<0.0001; Fig 3C).

**Fig 4:**
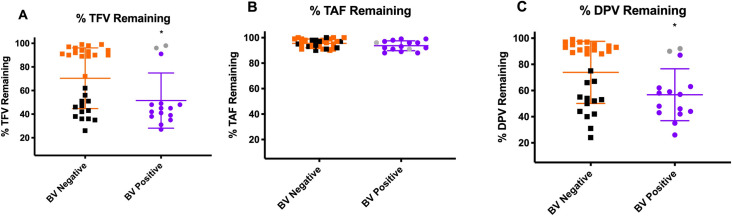
Fraction of drug remaining based on Nugent score diagnosed BV+/- women. (A) % TFV remaining after 24 hours at 37C in BV diagnosed vs non-BV diagnosed CVLs. Orange, BV negative; Purple, BV positive. Black dots indicate less than 50% *Lactobacillus* dominance despite BV negative diagnoses. Gray dots indicate greater than 50% *Lactobacillus* dominance despite BV positive diagnoses. (B) % TAF remaining after 24 hours at 37C in BV diagnosed vs non-BV diagnosed CVLs. Orange, BV negative; Purple, BV positive. Black dots indicate less than 50% *Lactobacillus* dominance despite BV negative diagnoses. Gray dots indicate greater than 50% *Lactobacillus* dominance despite BV positive diagnoses. (C) % DPV remaining after 24 hours at 37C in BV diagnosed vs non-BV diagnosed CVLs. Orange, BV negative; Purple, BV positive. Black dots indicate less than 50% *Lactobacillus* dominance despite BV negative diagnoses. Gray dots indicate greater than 50% *Lactobacillus* dominance despite BV positive diagnoses.

Of importance, we compared vaginal dysbiosis measured by 16S rRNA sequencing to clinical BV diagnosis by Nugent score. Amongst CVLs collected from women without diagnosed BV, almost 45% (thirteen women out of twenty-nine) had less than 50% *Lactobacillus* (Fig 4) and instead had a much more diverse community profile. These samples also exhibited the most degradation amongst non-BV samples and matched the samples from BV-positive women without *Lactobacillus* dominance (Fig 4A–4C). Similarly, amongst CVLs collected from women with diagnosed BV, roughly 13% (two women out of fifteen) had *Lactobacillus* dominance (Fig 4) and were amongst the highest fraction of drug remaining within the BV group (Fig 4A–4C). These women matched degradation profiles similar to women without BV and had *Lactobacillus*-dominance. When ranked by Nugent score, we observed a statistically significant association where increasing Nugent score correlated with more TFV degradation (R^2^ = 0.763 p<0.0001, S1A Fig). Similarly, we observed a significant association with increasing DPV degradation and Nugent score (R^2^ = 0.831, p<0.0001, S1C Fig). Of note, due to lack of TAF degradation, there was no significant trend with Nugent score (S1B Fig). Of note, sub-analysis of all experiments did not show a significant difference when comparing HIV infected vs uninfected.

### Effect of drug metabolism by vaginal bacteria from CVLs on drug uptake

**Fig 5:**
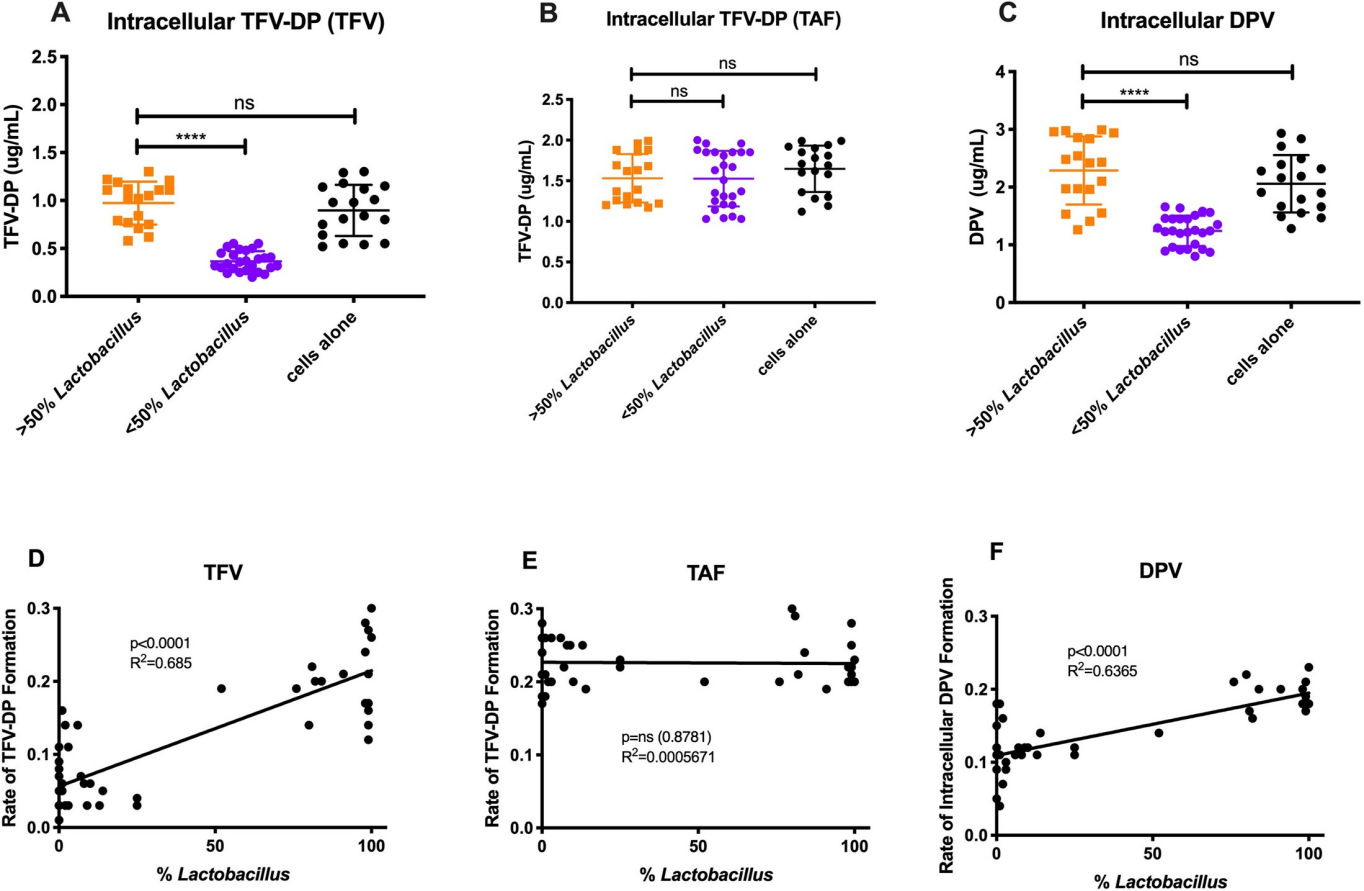
Intracellular active metabolite formation after 24 hours grouped by *Lactobacillus* dominance or non-dominance. **(A)** Amount of intracellular TFV-DP formed post-TFV administration after 24 hours at 37C in *Lactobacillus* dominant versus non-*Lactobacillus* dominant CVLs. Orange, greater than 50% dominance; Purple, less than 50% dominance. (B) Amount of intracellular TFV-DP formed post-TAF administration after 24 hours at 37C in *Lactobacillus* dominant versus non-*Lactobacillus* dominant CVLs. Orange, greater than 50% dominance; Purple, less than 50% dominance. (C) Amount of intracellular DPV formed post-DPV administration after 24 hours at 37C in *Lactobacillus* dominant versus non-*Lactobacillus* dominant CVLs. Orange, greater than 50% dominance; Purple, less than 50% dominance. (D) Rate of TFV-DP formation (amount/hour) vs. %*Lactobacillus* in CVLs after 24-hour incubation with TFV. Rate of degradation calculated as amount formed/24 hours. (E) Rate of TFV-DP formation (amount/hour) vs. %*Lactobacillus* in CVLs after 24-hour incubation with TAF. Rate of degradation calculated as amount formed/24 hours. (F) Rate of DPV formation (amount/hour) vs. %*Lactobacillus* in CVLs after 24-hour incubation with DPV. Rate of degradation calculated as amount formed/24 hours.

To determine if metabolism of vaginal bacteria alters drug uptake into target cells, we assessed intracellular pharmacologically active drug compounds from the Jurkat cell pellets after centrifugation of samples. Cell pellets were re-suspended in 1mL of R10 media, and a 10 uL aliquot was taken for cell counting. For TFV and TAF, we assessed intracellular TFV-DP, and for DPV, we assessed intracellular DPV, normalized for differences in cell counts. We evaluated the amount of active drug compounds in *Lactobacillus*-dominant versus non-*Lactobacillus* dominant samples. In *Lactobacillus*-dominant samples, we observed 0.973±0.224ug/mL of TFV-DP versus only 0.367±0.104ug/mL of TFV-DP in non-*Lactobacillus* dominant samples (p<0.0001) from TFV administration after 24 hours (Fig 5A). In negative control Jurkat cells + TFV alone, we saw 0.897±0.267ug/mL of TFV-DP formed, which was not statistically significant from *Lactobacillus* dominant samples (p>0.9999, Fig 5A). The difference in TFV-DP formation from TAF administration was not significantly different (p>0.9999) with an average of 1.53±0.298ug/mL versus 1.53±0.341ug/mL for *Lactobacillus* and non-*Lactobacillus* dominant samples, respectively (Fig 5B). In the negative control group containing Jurkat cells and TAF alone, we measured 1.646±0.285ug/mL of TFV-DP formed, which was not statistically significantly different from either experimental group (p>0.9999). In samples with DPV, intracellular DPV was significantly higher in *Lactobacillus* dominant samples versus non-*Lactobacillus* dominant samples (p<0.0001; Fig 5C). The median amount of intracellular DPV was 2.288±0.590ug/mL in *Lactobacillus* dominant samples versus only 1.241±0.263ug/mL in non-*Lactobacillus* dominant samples. The negative control group, Jurkat cells alone + DPV, demonstrated intracellular levels of DPV at 2.057±0.497ug/mL, which was not statistically significantly different from the *Lactobacillus* dominant samples (p>0.9999). When evaluating the intracellular formation of TFV-DP from TFV, we saw a significant correlation between active drug formation in cells and abundance of *Lactobacillus* (R^2^ = 0.685, p<0.0001; Fig 5D). Similarly, the presence of intracellular DPV exhibited a significant correlation between active drug formation and *Lactobacillus* abundance (R^2^ = 0.6365, p<0.0001; Fig 5F). However, the rate of TFV-DP formation after TAF administration did not correlate with *Lactobacillus* abundance (R^2^ = 0.0005671, p = 0.8781; Fig 5E). These data demonstrate that target (Jurkat) cells uptake the PrEP drugs TFV and DPV more efficiently in the presence of *Lactobacillus* with decreased PrEP uptake in non-*Lactobacillus* communities.

### The effect of vaginal microbiota on uptake efficiency of ART drugs

**Fig 6:**
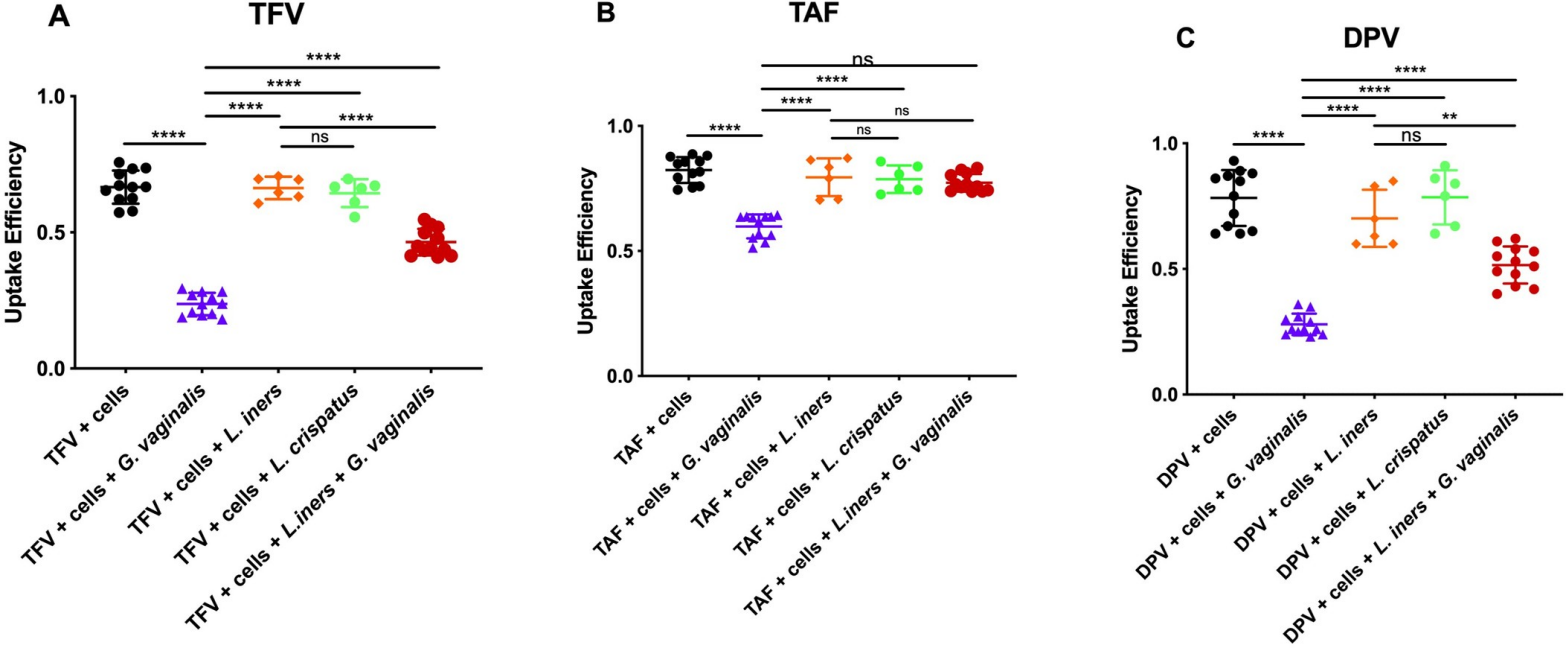
Uptake efficiency of Jurkat cells in converting drug into active metabolite after 1 hour. **Uptake efficiency is defined as the amount of intracellular drug divided by the amount of drug added extracellularly.** (A) Uptake efficiency of Jurkat cells in converting TFV into TFV-DP based on 1) TFV + cells alone (black) 2) TFV + cells + *G*. *vaginalis* (purple) 3) TFV + cells + *L*. *iners* (orange) 4) TFV + cells + *L*. *crispatus* (green) 5) TFV + cells + *L*. *iners* + *G*. *vaginalis* (red). Bacteria were added 15 minutes prior to incubation at 37C and added at a ratio of bacteria to cells of 2.5:1. Co-culture experiments (4) were added at a 1:1 ratio of *L*. *iners*: *G*. *vaginalis* and an overall ratio of total bacteria to cells at 2.5:1. N = 12. (B) Uptake efficiency of Jurkat cells in converting TAF into TFV-DP based on 1) TAF + cells alone (black) 2) TAF + cells + *G*. *vaginalis* (purple) 3) TAF + cells + *L*. *iners* (orange) 4) TFV + cells + *L*. *crispatus* (green) 5) TFV + cells + *L*. *iners* + *G*. *vaginalis* (red). Bacteria were added 15 minutes prior to incubation at 37C and added at a ratio of bacteria to cells of 2.5:1. Co-culture experiments (4) were added at a 1:1 ratio of *L*. *iners*: *G*. *vaginalis* and an overall ratio of total bacteria to cells at 2.5:1. N = 12. (C) Uptake efficiency of Jurkat cells in accumulating intracellular DPV based on 1) DPV + cells alone (black) 2) DPV + cells + *G*. *vaginalis* (purple) 3) DPV + cells + *L*. *crispatus* (orange) 4) TFV + cells + *L*. *crispatus* (green) 5) TFV + cells + *L*. *iners* + *G*. *vaginalis* (red). Bacteria were added 15 minutes prior to incubation at 37C and added at a ratio of bacteria to cells of 2.5:1. Co-culture experiments (4) were added at a 1:1 ratio of *L*. *crispatus*: *G*. *vaginalis* and an overall ratio of total bacteria to cells at 2.5:1. N = 12.

Given the decreased TFV and DPV levels after incubation with CVLs from women with non-*Lactobacillus* dominant microbiomes, we aimed to determine interactions between major vaginal bacterial taxa that may contribute to drug degradation. Using an *in vitro* co-culture system, we assessed potential bacteria-mediated metabolism using *Gardnerella vaginalis*, *Lactobacillus iners*, and *Lactobacillus crispatus*. Despite *Gardnerella vaginalis* not being the predominant taxa in the non-*Lactobacillus* samples, previous studies have identified these taxa amongst African women playing a major role in bacteria-mediate drug metabolism [4]. We found that in culture with *G*. *vaginalis*, there was a significant reduction in the amount of intracellular active drug formed versus drug administered (uptake efficiency) when compared with cultures with *Lactobacillus* spp. after 1 hour (p<0.0001; Fig 6A). Of note, when co-cultured together, the negative effects of *G*. *vaginalis* are somewhat ameliorated by the presence of *Lactobacillus*, albeit still significantly lower than *Lactobacillus* alone (p<0.0001). This addition of *Lactobacillus* does increase the efficiency where it is greater than *G*. *vaginalis* alone (p<0.0001). When comparing the uptake efficiency after TAF administration, *G*. *vaginalis* had the greatest negative impact when compared with controls (p<0.0001) and *Lactobacillus* spp. (p<0.0001). However, intriguingly, when co-cultured with both *G*. *vaginalis* and *Lactobacillus* spp. together, TAF degradation was no longer observed, and there is no statistically significant difference when compared with *Lactobacillus* spp. alone (p = 0.8143) and controls (p = 0.0780; Fig 6B). The presence of *G*. *vaginalis* also caused a significant reduction in uptake efficiency of DPV when compared with controls and *Lactobacillus* spp. alone (p<0.0001; Fig 6C). When co-cultured with both *Lactobacillus* and *G*. *vaginalis*, there was a significant increase in DPV uptake efficiency when compared with *G*. *vaginalis* alone (p<0.0001), but still significantly lower than *Lactobacillus* alone (p<0.0001). We did not see any observable difference uptake efficiency with any drug when co-cultured with *L*. *iners* vs. *L*. *crispatus* (Fig 6). Although we observed less formation of TFV-DP from TAF administration in cultures with *G*. *vaginalis*, the reason we do not see any changes in TFV-DP production amongst the *non-Lactobacillus* dominant samples is believed to be due to the relative abundance of *G*. *vaginalis*. That is, amongst the study samples, *G*. *vaginalis* was not the dominating taxa, while the in-vitro co-cultures strictly used *G*. *vaginalis*.

### HIV infection *in vitro* is increased in the presence of dysbiotic vaginal bacteria

**Fig 7:**
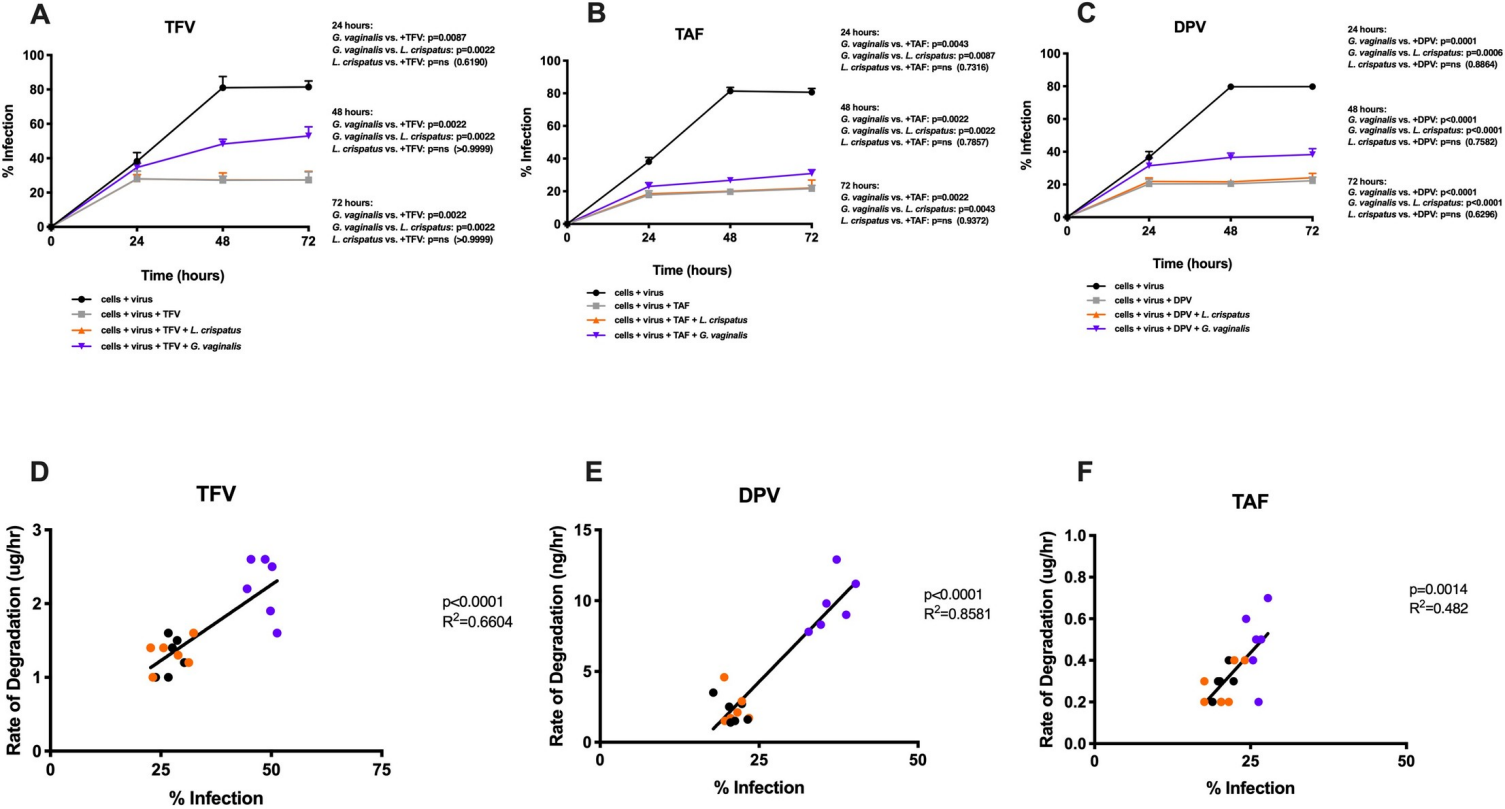
HIV-1_LAI_ infection of CEM-GFP cells after incubation at 37C. (A). % Infection of CEM-GFP cells over time up to 72 hours post-incubation with 1) CEM-GFP cells + HIV-1_LAI_ 2) CEM-GFP cells + HIV-1_LAI_ + TFV 3) CEM-GFP cells + HIV-1_LAI_ + TFV + *L*. *crispatus* 4) CEM-GFP cells + HIV-1_LAI_ + TFV + *G*. *vaginalis*. CEM-GFP cells were incubated for 30 minutes with bacterial inoculum. TFV was added and allowed to incubate for 1.5 hours prior to HIV-1_LAI_ addition. Purple *G*. *vaginalis*; Orange, *L*. *crispatus;* Black, virus alone; Gray, virus and TFV. N = 6. (B) % Infection of CEM-GFP cells over time up to 72 hours post-incubation with 1) CEM-GFP cells + HIV-1_LAI_ 2) CEM-GFP cells + HIV-1_LAI_ + TAF 3) CEM-GFP cells + HIV-1_LAI_ + TAF + *L*. *crispatus* 4) CEM-GFP cells + HIV-1_LAI_ + TAF + *G*. *vaginalis*. CEM-GFP cells were incubated for 30 minutes with bacterial inoculum. TAF was added and allowed to incubate for 1.5 hours prior to HIV-1_LAI_ addition. Purple *G*. *vaginalis*; Orange, *L*. *crispatus;* Black, virus alone; Gray, virus and TAF. N = 6. (C) % Infection of CEM-GFP cells over time up to 72 hours post-incubation with 1) CEM-GFP cells + HIV-1_LAI_ 2) CEM-GFP cells + HIV-1_LAI_ + DPV 3) CEM-GFP cells + HIV-1_LAI_ + DPV + *L*. *crispatus* 4) CEM-GFP cells + HIV-1_LAI_ + DPV + *G*. *vaginalis*. CEM-GFP cells were incubated for 30 minutes with bacterial inoculum. DPV was added and allowed to incubate for 1.5 hours prior to HIV-1_LAI_ addition. Purple *G*. *vaginalis*; Orange, *L*. *crispatus;* Black, virus alone; Gray, virus and DPV. N = 6. (D) Rate of TFV degradation versus % infection of CEM-GFP cells 48 hours after incubation with virus. Purple *G*. *vaginalis*; Orange, *L*. *crispatus;* Black, TFV alone. N = 6. (E) Rate of TAF degradation versus % infection of CEM-GFP cells 48 hours after incubation with virus. Purple *G*. *vaginalis*; Orange, *L*. *crispatus;* Black, TAF alone. N = 6. (F) Rate of DPV degradation versus % infection of CEM-GFP cells 48 hours after incubation with virus. Purple *G*. *vaginalis*; Orange, *L*. *crispatus;* Black, DPV alone. N = 6.

Given our findings that dysbiotic vaginal bacteria such as *G*. *vaginalis* can differentially impact drug concentrations, we investigated the impact of this phenomenon on HIV productive infection. Using CEM-GFP cells and HIV-1_LAI_ virus, we assessed productive infection of cells following cultures with drug as well as different bacteria. We monitored the fraction of CEM-GFP cells infected from 24 to 72 hours post-inoculation (Fig 7A). To determine if cells were infected, we used flow cytometry to measure GFP fluorescence. S2 Fig demonstrates representative staining plots of cells alone (A), cells + virus (B), cells + virus + TFV (C), cells + virus + TFV + *L*. *crispatus* (D), and cells + virus + TFV + *G*. *vaginalis* (E) looking at %GFP versus forward scatter. Gating strategy followed doublet exclusion and live dead analysis.

**Fig 8:**
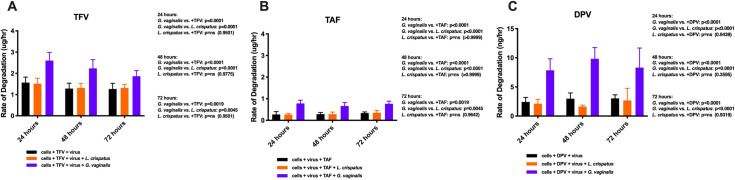
Degradation kinetics over time. (A) Rate of TFV degradation over time up to 72 hours. Purple *G*. *vaginalis*; Orange, *L*. *crispatus;* Black, TFV alone. N = 6. (B) Rate of TAF degradation over time up to 72 hours. Purple *G*. *vaginalis*; Orange, *L*. *crispatus;* Black, TAF alone. N = 6. (C) Rate of DPV degradation over time up to 72 hours. Purple *G*. *vaginalis*; Orange, *L*. *crispatus;* Black, TAF alone. N = 6.

In co-cultures, we saw a lower fraction of infected cells following TFV administration (5uM) when compared with virus and cells alone (p = 0.0379) and we observed no difference when compared with TFV and *Lactobacillus* (p = 0.6190) as early as 24 hours post-incubation (Fig 7A). While we saw no statistically significant difference between TFV administration alone and TFV with *G*. *vaginalis* (p = 0.1909), there was still a difference in median values (26.4 vs. 41.5). At 48 hours, we observed a significant difference in infected cells when co-cultured with *G*. *vaginalis* compared to TFV alone (p = 0.0022) and with TFV + *Lactobacillus* (p = 0.0022), and these trends continued through 72 hours (Fig 7A). To elucidate the effects of drug degradation on frequency of infected cells, we evaluated the correlation between rate of TFV degradation (μg/hour) with frequency of infected cells and observed a significant correlation at 48 hours (R^2^ = 0.6604, p<0.0001; Fig 7B). Fig 8A details the rate of degradation of TFV over all time points. Across all time points, we observed statistically significant difference between *G*. *vaginalis* and *Lactobacillus* or TFV alone (Fig 8). We did not see a significant difference between samples with *Lactobacillus* and abiotic controls.

Following TAF administration (5uM), there was a significantly lower amount of infected cells (p<0.0001) but no observable difference when *Lactobacillus* was added (p = 0.7316; Fig 7B). However, we saw a higher number of infected cells when cultured with *G*. *vaginalis* when compared with *L*. *crispatus* (p = 0.0087) after 24 hours. The 48 and 72-hour time points exhibited similar trends. Although significantly lower than the effects seen with TFV, we observed a significant correlation between the rate of TAF degradation and % of infected cells (R^2^ = 0.482, p = 0.0014; Fig 7E). Fig 8B summarizes the rate of TAF degradation over time. Across all time points, we observed statistically significant difference between *G*. *vaginalis* and *Lactobacillus* or TAF alone. We did not see a significant difference between samples with *Lactobacillus* and abiotic controls. However, despite this difference, overall, TAF had minimal degradation.

Finally, we evaluated the effects that vaginal bacteria had on the ability of DPV to prevent HIV infection *in vitro*. As early as 24 hours post-incubation, we observed a significant difference in infected cells when compared with controls (p<0.0001; Fig 7C). DPV was added at 5uM. Similar to TFV and TAF, we saw no difference between DPV alone and DPV with *Lactobacillus* (p = 0.8864; Fig 7C). We saw a higher frequency of infected cells when cultured with *G*. *vaginalis* compared with DPV alone (p = 0.0001) and when compared with DPV + *Lactobacillus* (p = 0.0006). We also found a significant correlation between rate of DPV degradation and frequency of infected cells (R^2^ = 0.8581, p<0.0001; Fig 7F). Lastly, Fig 8C depicts the rate of DPV degradation over time. Across all time points, we observed a statistically significant difference between *G*. *vaginalis* and *Lactobacillus* or DPV alone (Fig 8C). We did not see a significant difference between samples with *Lactobacillus* and abiotic controls.

In samples with TFV concentrations > 15uM, the percentage of infected cells did not significantly change, implying that infected cells did not produce new infectious virions at these drug concentrations. However, in samples <5uM, the number of fluorescent cells increased with time for up to 48 hours, indicating viral escape and infection of new target cells. These data match IC50 values for TFV [27]. Of note, at high MOIs (>0.1), a decrease in fluorescence was observed at later time points (post 72 hours), because infected CEM-GFP cells are killed by virus-induced apoptosis [28]. Experiments using TAF and DPV yielded similar results.

### TFV uptake into Jurkat cells and *G*. *vaginalis* is inhibited by endocytosis inhibitors

**Fig 9:**
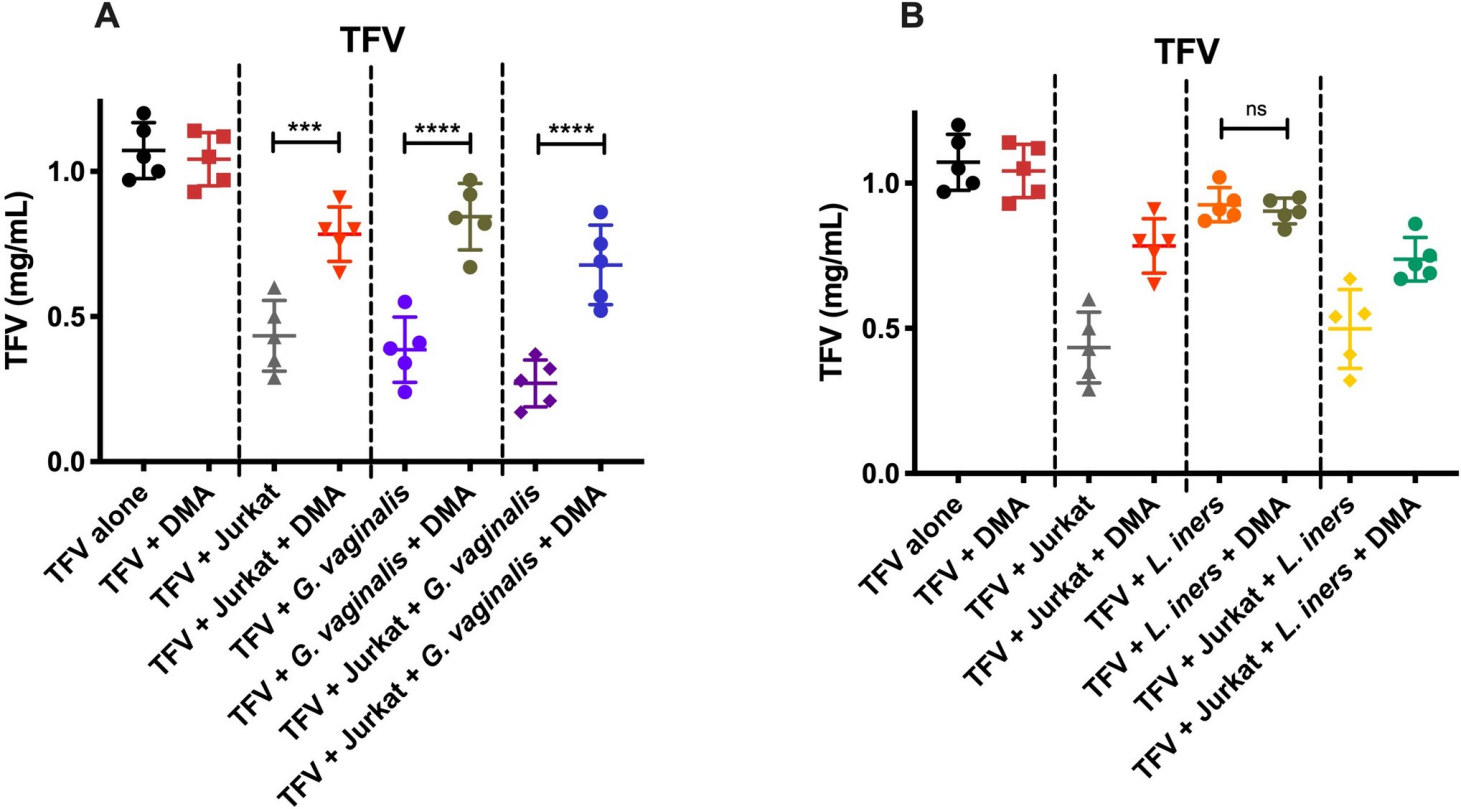
TFV remaining after Incubation with endocytosis inhibitor, DMA. (A) TFV remaining after 24 hours based on 1) TFV alone (black) 2) TFV + DMA (red) 3) TFV + Jurkat (gray) 4) TFV + Jurkat + DMA (dark orange) 5) TFV + *G*. *vaginalis* (purple) 6) TFV + *G*. *vaginalis* + DMA (dark green) 7) TFV + Jurkat + *G*. *vaginalis* (dark purple) 8) TFV + Jurkat + *G*. *vaginalis* + DMA (dark blue). Jurkat cells were exposed to 100uM DMA for 30 min prior to treatment with TFV (1mg/mL) and *G*. *vaginalis*. Co-culture experiments were added at a 2.5:1 ratio of bacteria to cells. N = 5. (B) TFV remaining after 24 hours based on 1) TFV alone (black) 2) TFV + DMA (red) 3) TFV + Jurkat (gray) 4) TFV + Jurkat + DMA (dark orange) 5) TFV + *L*. *iners* (orange) 6) TFV + *L*. *iners* + DMA (dark green) 7) TFV + Jurkat + *L*. *iners* (yellow) 8) TFV + Jurkat + *L*. *iners* + DMA (teal). Jurkat cells were exposed to 100uM DMA for 30 min prior to treatment with TFV (1mg/mL) and *L*. *iners*. Co-culture experiments were added at a 2.5:1 ratio of bacteria to cells. N = 5.

Given our findings, we investigated potential uptake mechanisms to elucidate how *G*. *vaginalis* metabolize TFV. Using Jurkat cells, we assessed extracellular TFV levels after 24 hours of co-culture with different bacteria. We monitored TFV levels after co-culture with dimethyl amiloride (DMA), a known NA^+^-H^+^ exchanger inhibitor, in the presence of Jurkat cells only, *G*. *vaginalis only*, and Jurkat cells with *G*. *vaginalis* (Fig 9). Similar experiments were done using *L*. *iners* (Fig 9). Following the administration of DMA, we saw an increase in extracellular TFV in the presence of Jurkat cells when compared with Jurkat cells alone (p = 0.0003, Fig 9A). Similarly, the addition of DMA also increased extracellular TFV levels in the presence of *G*. *vaginalis* when compared with *G*. *vaginalis* alone (p<0.0001, Fig 9A). Together, the effects of Jurkat cells and *G*. *vaginalis* on extracellular TFV levels were negated with the addition of DMA (p<0.0001, Fig 9A). Conversely, the administration of DMA to *L*. *iners* co-cultures had no impact on extracellular TFV levels when compared with *L*. *iners* alone (p>0.9999, Fig 9B).

### Ordinary Differential Equations (ODE) modeling of microbial metabolism provides insight to specific mechanistic components that mediate uptake efficiency

**Fig 10:**
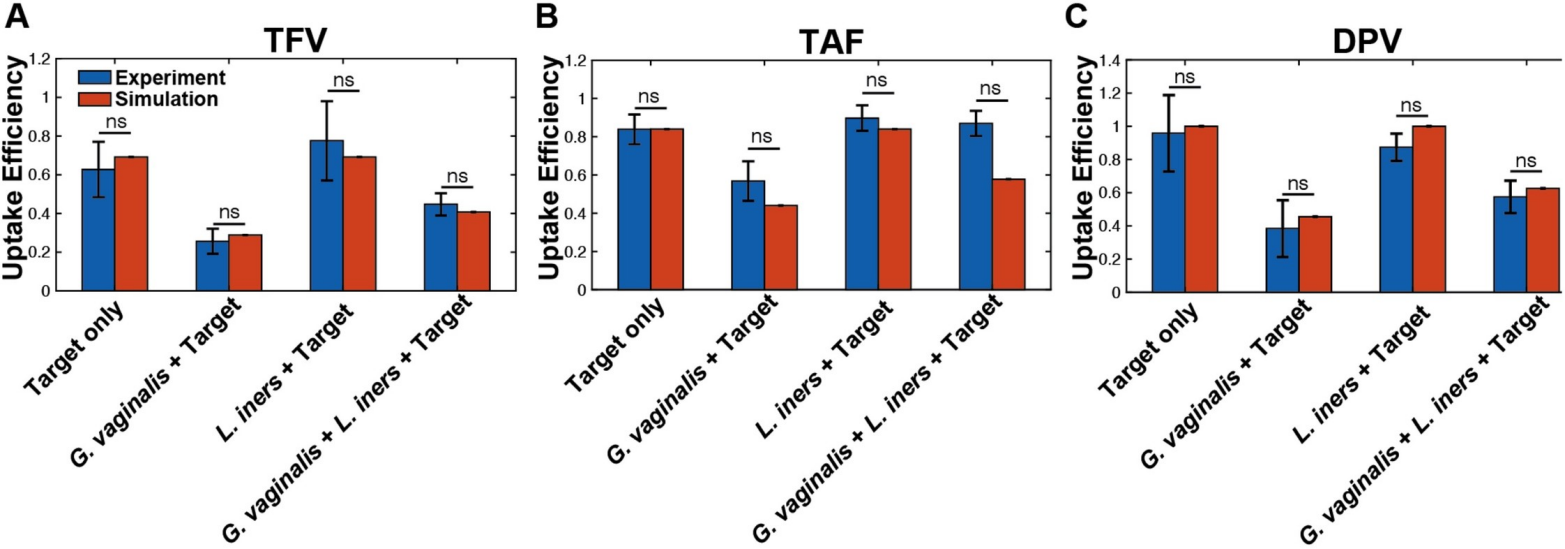
Validation of ODE model of ART drug kinetics. Comparison of experimental data (blue) to model-predicted uptake efficiency (orange) of Jurkat cells in converting. (A) TFV to TFV-DP based; (B) TAF to TFV-DP; and (C) DPV based on 1) TFV + cells alone 2) TFV + cells + *G*. *vaginalis* 3) TFV + cells + *L*. *iners* 4) TFV + cells + *L*. *iners + G*. *vaginalis* (ns denotes p > 0.05). Uptake efficiency is defined as the amount of intracellular drug divided by the amount of drug added extracellularly.

To understand the interaction between bacteria and target cells on the uptake efficiency of each drug, we developed a mathematical model that analyzes the competition between *G*. *vaginalis*, *L*. *iners* and target cells for drug internalization. The model assumes that the relative population of *G*. *vaginalis* to *L*. *iners* dictates the amount of drug internalized by Jurkat cells that can subsequently undergo conversion to active forms. Notably, when we compared the predicted uptake efficiency of for TFV, TAF and DPV for Drug + cells, Drug + cells + *G*. *vaginalis*, Drug + cells + *L*. *iners*, drug + cells + *L*. *iners* + *G*. *vaginalism*, model results were not significantly different than experimental results (Fig 10).

TFV + cells + *L*. *crispatus* was not explicitly determined, but since *L*. *iners* and *L*. crispatus do not significantly internalize TFV, DPV, or TAF, the predictions for both bacterial species would be mathematically equivalent. The prediction that *L*. *iners* and *L*. *crispatus* co-cultured with target cells would exhibit comparable uptake efficiencies for each drug was observed experimentally (Fig 6). These results suggest that the quantity of bacteria able to internalize and metabolize the drug relative to the quantity of bacteria unable to interact with the drug significantly dictates the amount of active drug formed.

Notably, both experimentally and in model predictions, TAF was the only drug that has comparable uptake efficiency between TAF + cells + *L*. iners + *G*. *vaginalis* with controls and cultures with only Lactobacillus spp. Since the model structure is equivalent across all drugs and input bacterial combinations, the only components of the system that could be responsible for this response are the model parameters such as rate of internalization into target or *G*. *vaginalis* (S2 Table). While internalization rates of TAF into target cells or *G*. *vaginalis* was not statistically different from TFV or DPV rates, TAF had the largest relative ratio of target internalization rate to *G*. *vaginalis* internalization rate compared to TFV and DPV (p = 0.0069; 0.0113; S2 Table). The faster internalization rate of TAF into target cells relative to *G*. *vaginalis* drug internalization rate could explain why TAF is less sensitive to *G*. *vaginalis* mediated metabolism, as the target cells outcompete *G*. *vaginalis* to interact with the drug.

**Fig 11:**
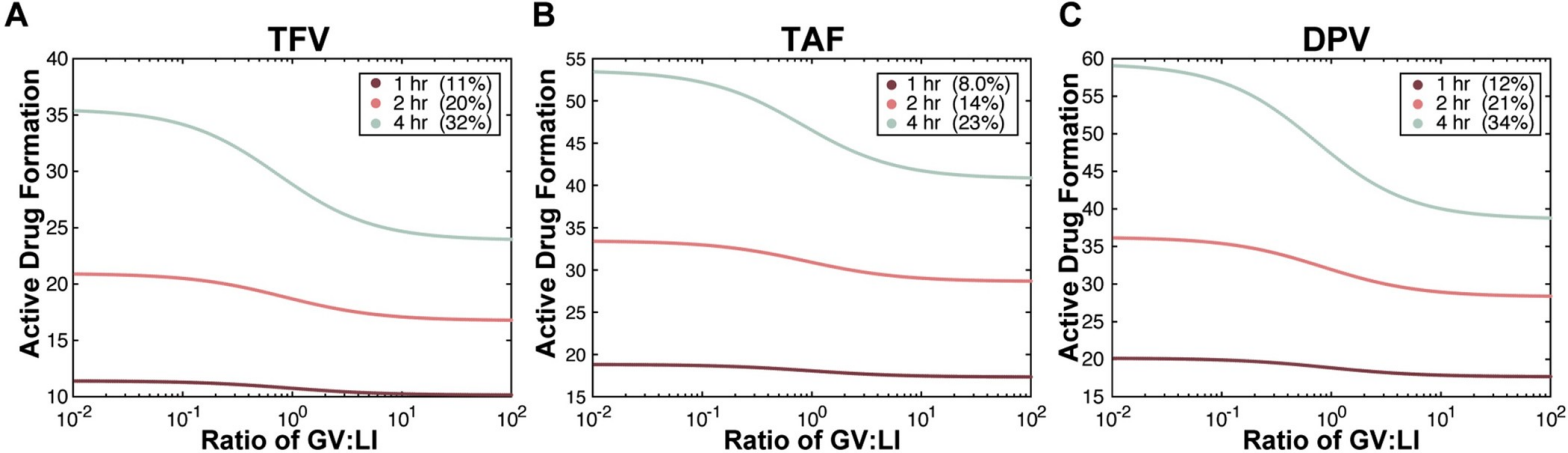
Time after MNZ treatment magnifies the effect of *G*. *vaginalis* on active drug formation. Simulations of active drug formation after 1hr, 2hr and 4hr post-treatment with MNZ at varying relative amounts of *G*. *vaginalis* to *L*. *iners* (GV:LI ratio) from 0.01-fold less *G*. *vaginalis* to *L*. *iners* to 100-fold more *G*. *vaginalis* to *L*. *iners* for (A) TFV; (B) TAF; and (C) DPV. The percent change from 0.01-fold GV:LB to 100-fold GV:LB active drug concentrations is listed in the legend.

To explore the relationship between microbiota composition and active drug formation beyond what was measured experimentally, we simulated varying the ratio of bacteria that can metabolize these drugs to bacteria that are unable to metabolize these drugs using the rates parameterized for *G*. *vaginalis*. Again, TFV and DPV exhibited more prominent bacteria-mediated degradation, and therefore there was an apparent decrease in active drug formation for ART drugs compared to TAF (Fig 11). Fascinatingly, the effects of varying microbial populations had greater influences on active drug formation as the time from drug administration increased, suggesting time between doses could be even more important for patients with vaginal dysbiosis.

## Discussion

This study provides the first detailed characterization of the impact of the vaginal microbiome on antiretroviral drug PK/PD using both *ex vivo* and *in vitro* assays. Using primary CVL samples, we confirmed that TFV is metabolized by vaginal bacteria from women with dysbiosis and demonstrated for the first time a decrease of DPV in the presence of vaginal dysbiosis. Importantly, we describe that TAF does not exhibit prominent bacteria-mediated degradation.

Here we calculated the degradation efficiency, defined as the amount of drug lost per hour. The inverse relationship of the relative abundance of *Lactobacillus* and the lower the rate of TFV and DPV degradation emphasizes the importance of *Lactobacillus* in preventing microbe drug degradation. While drug levels do highlight the potential for these interactions, in order to elucidate the effect on pharmacodynamically active metabolites, we assessed intracellular active drug levels. *Lactobacillus* dominance impacts extracellular and intracellular TFV and DPV concentrations. As a result, the amount of *Lactobacillus* also impacts the formation of intracellular TFV-DP and the accumulation of intracellular DPV, respectively. Rates of intracellular formation also correlate with how much *Lactobacillus* is present, further emphasizing the importance of *Lactobacillus* dominance. The data also support that TAF conversion to TFV-DP is mostly unaffected by vaginal microbiota, potentially due to more rapid uptake mechanisms than TFV or DPV, however further studies are needed to assess the precise mechanisms underlying the observed differences.

Taken in this context, the results indicate an additional explanation for low PrEP efficacy in trials in women. While high adherence is certainly critical to protection, the studies here suggest that lack of effectiveness and lower drug levels may be, at least in part, driven by vaginal microbial communities. Despite a recent analysis of the Partners PrEP study demonstrating no difference in oral PrEP efficacy in BV versus non-BV women defined by Nugent score [29], this study identifies a gap in accurate diagnosis and how “molecular BV”, or BV as identified using 16S rRNA sequencing to delineate specific bacteria [30], is likely a better predictors of bacteria-mediated metabolism than by using Nugent score only (S3 Fig). Furthermore, a recent study [31] highlighted production of TFV-DP from *L*. *crispatus*, however we saw no TFV-DP formation when cultured with TFV alone and attribute this difference to lack of direct measurements of TFV, DPV or TFV-DP in the Taneva et al. study [31]. To measure their TFV-DP levels from Jurkat cells, this group does not use LC-MS/MS but rather liquid scintillation counting, which only measures radioactivity. If a metabolite were being broken down extracellularly or transported out of the bacteria cell upon bacteria mediated metabolism, the radioactivity would measure the same. Thus, while this study attributed the decreases in PrEP drug levels they observed to binding rather than metabolism, they did not directly measure drug levels nor metabolism, and thus the highly comprehensive pharmacokinetic studies here demonstrate that metabolism is the more likely mechanism for PrEP drug loss in the presence of dysbiotic microbes. However, whichever mechanism, the vaginal microbiome is clearly critical for PrEP drug efficacy, and using drugs that are not subject to metabolism by the vaginal microbiome, such as we demonstrate here with TAF, is critical for future trials. However, further studies are needed to elucidate the discrepancies between Nugent score and 16S rRNA sequencing, and to improve diagnostic tools of BV in clinical practice and to predict the efficacy of PrEP.

While the prevalence of *Lactobacillus* dominance seems to be the major driving factor in bacteria-mediate metabolism, our *in vitro* cultures also elucidated specific bacterial species with efficiency of conversion to active drug compounds. *G*. *vaginalis* has been previously shown to cause biodegradation in samples from women who participated in the CAPRISA 004 trial [4] and here, significantly alters the efficiency in TFV, TAF, and DPV. Of note, TAF uptake efficiency in the presence of *G*. *vaginalis* was not significantly different than the uptake efficiency of TFV with cells alone, emphasizing the difference in uptake mechanisms exhibited by these two compounds. As previously demonstrated, TFV exhibits poor permeability into cell membranes and relies on an energy-dependent uptake mechanism mediated by uptake transporters or endocytosis [32]. Previous reports have identified organic anion transporters (OAT) 1 and 3 playing a key role in TFV uptake [32]. TAF is a newer TFV-based compound, approved for treatment of HIV and undergoing evaluation as a PrEP agent, characterized by rapid cell uptake. TAF is much more lipophilic than TFV due to the amidate group and relies primarily on passive diffusion, and is characterized by a rapid cell uptake [32]. The DMA studies highlight potential mechanisms into how TFV is uptaken into Jurkat and *G*. *vaginalis* cells. The use of an endocytosis inhibitor, DMA, prevented intracellular uptake into *G*. *vaginalis* highlighting a potential preferential targeting of TFV by *G*. *vaginalis*. This is a phenomenon not seen with *L*. *iners*. Further studies are needed to determine whether *G*. *vaginalis* uptake can be inhibited specifically, thus negating the effects caused by these dysbiotic microbes. In doing so, women with *G*. *vaginalis* may be protected against bacteria-mediated metabolism of TFV. Future studies should also include the use of other *Lactobacillus spp*. as well as other PrEP therapies.

Our *in vitro* studies also allowed us to explore potential therapeutic benefits as well as mimic samples from women who are transitioning from non-BV to BV. Our co-cultures with combinations of *Lactobacillus* spp. and *G*. *vaginalis* highlight the potential for a therapeutic benefit of administering *Lactobacillus* to women with BV to prevent these negative interactions with drugs. Utilizing novel *Lactobacillus* based live biotherapeutics such as Lactin V may improve efficacy of PrEP drugs in women [33]. Of note, when doing a mixed culture with TAF, we completely negate even the minor effects caused by *G*. *vaginalis*. It appears both healthy vaginal microbial communities and drug compound structure and uptake play a significant role in varying efficacy.

Our study not only has demonstrated *Lactobacillus* association with altering drug levels and impacting the formation of intracellular active metabolites, but also affecting productive infection of cells *in vitro*. Our infection assays further validated the impact *G*. *vaginalis* can have on productive cell infection as the drug metabolism effect is observed as soon as 24 hours and if 72 hours post-inoculation. We observed similar trends using DPV and with TAF supporting the notion that some BV-associated bacteria linked to bacterial-mediated drug metabolism have no preference of NRTIs over NNRTIs. Of note, we did not test TDF for metabolism due to the prodrug not being formulated for microbicide use. While TDF is a main component in oral PrEP, the relevance for vaginal microbes is not as pertinent as TFV, TAF, and DPV, all of which have been or are undergoing evaluation for topical use. However further studies related to gastrointestinal microbiome and oral PrEP drug metabolism are urgently needed.

With our novel ODE models, we illustrate how it is possible to confront the discrepancy of the apparent bacteria-mediated degradation of TAF between the *ex vivo* CVL samples and the *in vitro* samples by quantification of the rates at which strains other than *G*. *vaginalis* interact with TAF. Here, we were able to demonstrate that TAF has a faster rate of internalization in target cells relative to the internalization rate of TAF into *G*. *vaginalis* and that this quality of TAF leads to less sensitivity to *G*. *vaginalis-*mediated degradation of the drug. This framework can be modified to include additional microbes and it will allow for investigation of how other major BV-associated microbes degrade ART drugs and provide insight into whether PrEP drug therapy will be less effective dependent on personalized microbiota differences.

This study also demonstrates how critical it is to overcome the lack of effective treatments for BV. Metronidazole is the standard of care for clinically diagnosed BV, however has extremely high recurrence [30]. There are novel treatments that may promote a healthy, *Lactobacillus-*dominant vaginal microbiome such as Lactin V, a *Lactobacillus crispatus* live biotherapeutic that recently demonstrated efficacy in long term *Lactobacillus* restoration in a phase II clinical trial [33]. In addition, a recent pilot trial demonstrated that supplementing metronidazole with probiotic bacteria (either Ecologic Femi+ vaginal capsule (containing multiple *Lactobacillus* and one *Bifidobacterium* species), or Gynophilus LP vaginal tablet (*L*. *rhamnosus* 35)) for two months resulted in decreased polymicrobial anaerobic BV bacteria and increased *Lactobacillus* [34]. Furthermore, recent intriguing data demonstrated that vaginal microbiome transplantation from women with *Lactobacillus* dominance to women with BV could potentially be more efficacious than antibiotics alone and could provide another therapeutic option to more effectively treat BV and reduce STI and HIV infections [35].

Limitations of this study include the lack of longitudinal samples where shifts in bacterial communities could be monitored and assessed for corresponding drug level changes. Furthermore, the lack of availability of samples from clinical trials with DPV or TAF as a PrEP drug in women is a limitation. Additional studies quantifying bacterial concentrations via qPCR would also provide more information regarding these host-microbe drug interactions. Future studies should also include evaluating other BV-associated bacteria such as *Prevotella*, *Mobiluncus*, *Shuttleworthia*, and *Sneathia*. Our preliminary studies have demonstrated that many bacteria can biodegrade TFV and DPV *in vitro*, highlighting the importance of future studies using mixed cultures.

Taken together, these studies elucidate potential interactions between drugs and microbes, providing mechanisms for the variability seen in translating pharmacokinetics and pharmacodynamics in clinical trials, as well as highlighting the significant role the microbiome plays in drug uptake to target sites and systemic availability, and how this may affect virus transmission and treatment. Women are a highly underserved community when it comes to HIV prevention and treatment and are amongst the most vulnerable population for HIV acquisition. To better design and conduct clinical studies assessing HIV prevention in women, it is essential to better understand how the female reproductive tract microbiome plays a role in preventing therapeutic drug levels from being met. Furthermore, it is crucial that we are able to diagnose vaginal dysbiosis more rapidly and precisely, and to consider the microbiome as a factor in selecting the best prevention strategies for both men and women. Finally, it is imperative that we identify better interventions to improve the microbiome to be a more conducive environment for PrEP efficacy, which is critical for protection of women from STI and HIV transmission.

## Methods

### Ethics statement

All human studies were approved by the University of Miami Institutional Review Board (IRB) number 20130623, and written consent was obtained from all participants.

### Study procedures

The study utilized a cross sectional design, enrolling women with HIV or at risk for HIV infection, and it was conducted at the AIDS Clinical Research Unit at the University of Miami. The study was undertaken in cooperation with the Miami Women and HIV Interagency Study (WIHS) and the Miami Center for AIDS Research (CFAR). Eligibility criteria for participation were women 18 to 45 years of age and being sexually active in the three months previous to study enrollment. Women included also had no history of cervical dysplasia, were not taking immunosuppressant medications, and had no malignancies. To avoid the effect of hormones on the mucosa, pregnant women or women using contraceptive medications or intrauterine devices were excluded.

### HIV testing

The OraQuick ADVANCE Rapid HIV- 1/2 Antibody Test was used to determine HIV status for women without documentation of HIV status. Positive results were followed by a confirmatory HIV western blot. Participants known to be infected with HIV prior to the study, presented documentation of positive HIV status, such as HIV Western blot results, medical records, or two laboratory results with detectable HIV viral loads greater than 1000 copies/ml, and had a rapid test performed as confirmation.

### STI testing

Chlamydia and gonorrhea were tested in urine samples using nucleic acid amplification tests (NAAT). All women included were negative for these tests.

### Genital sample collection

FRT cervicovaginal lavages were collected by inserting a vaginal speculum and utilizing a procedure that has previously been described [36]. A vaginal swab was used Gram stained for Nugent scoring. Cervicovaginal lavage (CVL, 10 ml) was collected and immediately placed on ice and aliquoted and stored at -80°C. Samples were transported in dry ice.

### WIHS CVLs and PrEP drug experiments

CVLs were thawed and centrifuged at 4,000 RPM, 4C for 10 minutes and resuspended into 1.5mL fresh NYCIII medium. Each CVL was divided into 3x300uL aliquots. Each aliquot was labeled with the sample name and #1, #2, and #3, respectively. Each aliquot was allowed to rest in anaerobic conditions (80% N_2_, 10% CO_2_, 10% H_2_) for 4 hours prior to drug degradation experiments. Once thawed and rested, 5ug/mL of TFV (Selleckchem) was added to aliquot #1. Similarly, 5ug/mL of TAF (Selleckchem) and 5 ug/mL of DPV (Selleckchem) were added into aliquots #2 and #3, respectively. Once the drugs were added, HIV target cells (Jurkat, ATCC) were added at a concentration of 1x10^5^ cells into each aliquot. Samples were then incubated under anaerobic conditions for 24 hours. 50uL sample time points were taken at t = 0, t = 4 hours, and t = 24 hours. Samples were compared to just bacteria inoculum from CVLs and drug alone (no Jurkat cells) as well as bacteria inoculum alone (No Jurkat cells or drug). Samples were prepared for mass spectrometry and aliquots saved for microbiome analysis as described below.

### Bacterial vaginosis by Nugent criteria

Bacterial vaginosis was diagnosed by Gram staining techniques of slides with vaginal secretions at the University of Miami microbiology laboratories within 24 hours of collection and the same staff performed staining a reading for all the samples. Unclear cases were reviewed and confirmed by a second technician and the laboratory chief once a consensus was reached. The slides were judged based on Nugent criteria. A Nugent score of 7 or above was classified as bacterial vaginosis.

### ART Drug degradation kinetic experiments

Jurkat cells (ATCC clone ES-1TIB-152) were placed in cultures at 5x10^5^ cells/well in RPMI and were rested at 37C for 1 h. *L*. *iners*, *L*. *crispatus* or *G*. *vaginalis* were added at 2.5 bacteria cells/Jurkat cell, while drugs were added at 0.1 mg/mL. Supernatant was collected, and cells saved at time 0, 30, 60, and 120 minutes. Cells were separated from bacterial cells using Millenia Biotec CD3 Microbeads isolation kit. Samples were prepared and analyzed for mass spectrometry as described below.

### DMA inhibitor experiments

Jurkat cells (ATCC clone ES-1TIB-152) were placed in cultures at 2.5x10^5^ cells/well in RPMI and were exposed to DMA (100uM) at 37C for 30 minutes. *L*. *iners* and *G*. *vaginalis* were added at 2.5 bacteria cells/Jurkat cell, while drug levels were added at 1 mg/mL. Supernatant was collected, and cells saved at time 0 and 24 hours. Samples were prepared and analyzed for mass spectrometry as described below.

### Bacteria-drug HIV infection experiments

CEM-GFP cells are non-adherent and were obtained from the NIH AIDS Reagent Program and were cultured in RPMI 1640 medium with 10% fetal bovine serum, 1% penicillin-streptomycin, and 1% 200mM L-glutamine. HIV-1_LAI_ was titered by terminal dilution on CEM-GFP cells at different multiplicities of infection for 4 h at 37C in the presence of 2ug/mL polybrene. Cells were then subjected to a wash with sterile PBS 2x and re-suspended in 2mL culture medium without penicillin-streptomycin and incubated at 37C in 5% CO2. 200uL aliquots were taken daily for 6 days and fixed in 1% PFA prior to flow cytometry. CEM-GFP cells (2.5x10^5^) were incubated for 30 minutes with bacterial inoculum, then for 1.5 h at 37C with TFV, TAF, or DPV before infection at a MOI of 0.1 with a T-cell tropic strain of HIV-1_LAI_ (5x10^6^ TCID_50_/mL) [37]. An aliquot of 2x10^5^ cells for each condition was harvested daily for 4 days after infection. Cells were then subjected to an Aqua Live/Dead stain, washed with PBS and fixed with 1% PFA prior to flow cytometry analysis. Conditions included CEM-GFP cells alone, CEM-GFP cells + TFV, CEM-GFP cells + TAF, CEM-GFP cells + DPV, CEM-GFP cells + TFV + *G*. *vaginalis*, CEM-GFP cells + TAF + *G*. *vaginalis*, CEM-GFP cells + DPV + *G*. *vaginalis*, CEM-GFP cells + TFV + *L*. *iners*, CEM-GFP cells + TAF + *L*. *iners*, and CEM-GFP cells + DPV + *L*. *iners*. 200uL aliquots of sample media were also taken for drug degradation analysis using methods described below. Both bacteria did not alter their growth curves using the CEM-GFP media without penicillin-streptomycin.

### Analysis of 16S rRNA gene sequencing data

Bacteria from cervicovaginal lavage samples were pelleted by centrifugation and resuspended in 200μl of lysis buffer (30mM Tris-HCl, 10mM EDTA, 200mM sucrose, pH 8.2). Samples were heated at 65°C for 10 minutes prior the addition of 10mg/ml lysozyme solution. Samples were then incubated for 1 hour at 37C. 5% SDS was then added to a final concentration of 1% w/v and incubated at 56°C for 10 minutes. DNA extractions were then performed using Qiagen’s Dnaeasy Blood and Tissue Kit. Extracted DNA was amplified following the Earth Microbiome Protocol for 16S Illumina sequencing utilizing 515F-806R primers [38] to target the V4 region of the 16S SSU rRNA. Amplicon concentrations were normalized, pooled, and cleaned prior to KAPA quantification. The pooled library was sequenced using a 2x150 bp Illumina MiSeq run. 16S sequencing reads were processed using QIIME2 version 2018.2; taxonomic determination in QIIME2 utilized the Silva 119 classifier. Taxonomic plots were created in part within RStudio utilizing the phyllode package.

### ART concentrations by mass spectrometry

TFV, TAF, DPV, and TFV-DP concentrations were determined by validated LC-MS/MS assays. Drugs were extracted from media via protein precipitation with acetonitrile. Chromatographic separation was achieved using a gradient elution with a Chromolith Performance RP-C18 column. The column was maintained at 25°C throughout. Samples were subjected to positive electrospray ionization (ESI) and detected via multiple reaction monitoring (MRM) using an LC-MS/MS system (Agilent Technologies 6460 QQQ/MassHunter). Calibration standards were prepared with an inter- and intra-day precision and accuracy of ≤10.1% with an r^2^ value of 0.9981±0.0017. Quantification was performed using MRM of the transitions of m/z 288.1→176.1, 477.1→270.1, 330.4→158.1, 448.0→270.0, 294.1→182.1, and 343.4→171.1 for TFV, TAF, DPV, TFV-DP, TFV internal standard (IS) -d6, and DPV IS -d13 respectively. Each transition was monitored with a 150-ms dwell time. Stock solutions of TFV, TAF, DPV, TFV-DP, and both IS were prepared at 1mg/mL in acetonitrile-water and stored at -20°C. Mobile phase A is 0.1% acetic acid in H2O and mobile phase B is 0.1% acetic acid in ACN, and chromatographic separation was achieved using a gradient elution with a Chromolith Performance RP-C18 column from 0–4.6 minutes, B% 0–100, with 0.5 flow and 200 max pressure. During pre-study validation, calibration curves were defined in multiple runs based on triplicate assays of spiked media samples as well as QC samples. This method was validated for its sensitivity, selectivity, accuracy, precision, matrix effects, recovery, and stability. Degradation analysis was performed blinded, such that study samples were indistinguishable to individuals performing the experiments. Replicates of reference samples were included every 4 samples and evenly distributed throughout the MS analysis to monitor consistency and performance and to utilize for downstream normalization.

### Bacterial strains and culture conditions

*Lactobacillus iners* ATCC 55195, *Lactobacillus crispatus*, ATCC 33197, and *Gardnerella vaginalis* ATCC 14018 (group C) were obtained from the American Type Culture Collection (ATCC) and maintained on Human Bilayer Tween Agar (BD) plates and New York City III (NYCIII) medium according to the manufacturer’s instructions. Agar plates and liquid cultures were incubated at 37C with 5% CO_2_ atmosphere. Liquid cultures were incubated at 37C in aerobic conditions. Frozen stocks of strains were stored at -80C in 40% (v/v) glycerol.

### ART drug kinetics mathematical model

A system of ordinary differential equations (ODEs) was developed to model the drug degradation kinetics exhibited by *G*. *vaginalis* on TFV, TAF and DPV. Inputs into the model included the cell count of *L*. *iners*, cell count of *G*. *vaginalis* and cell count of Jurkat cells. The outputs included the concentration of external drug, concentration of internal drug in target cells, active drug formed, and adenine formation in *G*. *vaginalis*. Parameters were derived from the ART Drug degradation kinetic experimental data for individual cultures of *L*. *iners*, *G*. *vaginalis* and Jurkat cells. The internalization rate of TFV, TAF and DPV into *G*. *vaginalis*, *L*. *iners* and Jurkat cells was determined by fitting the experimental kinetic data to the system of ordinary differential equations using lsqcurvefit (MATLAB) and multistart (MATLAB) to determine global solutions. The rate of TFV and TAF conversion to TFV-DP was determined using the kinetic experiments in cultures with only target cells by the same methodology.

### Statistical analysis

GraphPad Prism statistical software (version 6; GraphPad Software, San Diego, CA) was used for all statistical analyses. Results obtained at the pre-incubation time point were compared with those obtained at 24 hours for the WIHS CVL samples. Similarly, in vitro experiments with Jurkat cells compared pre and 1-hour post-incubation time points. CEM-GFP experiments were compared between pre-incubation and 24, 48, and 72 hours post-incubation. The significance between time points was evaluated using Mann-Whitney tests. *P* values of <0.05 were considered significant. Rates of degradation versus *Lactobacillus*, formation of metabolites versus *Lactobacillus*, and rates of degradation versus infection were calculated using linear regression analysis. The goodness of fit was evaluated using R square as well as *P* values to assess non-zero slope significance. To generate relative abundance plots, we focused on the top 19 abundant genera. The significance between multiple experimental conditions was evaluated using one-way ANOVA Tukey’s multiple comparison test. *P* values of <0.05 were considered significant.

## Supporting information

S1 Fig Rate of PrEP drug degradation relative to nugent score. (A) Rate of TFV degradation vs. Nugent score in CVLs after 24-hour incubation with DPV. Rate of degradation calculated as amount lost/24hours. (B) Rate of TAF degradation vs. Nugent score in CVLs after 24-hour incubation with DPV. Rate of degradation calculated as amount lost/24hours. (C) Rate of DPV degradation vs. Nugent score in CVLs after 24-hour incubation with DPV. Rate of degradation calculated as amount lost/24hours.
(TIF)

S2 Fig Representative staining plots demonstrating % fluorescence of cem-gfp cells 48 hours after 37c incubation. (A) % GFP fluorescence of CEM-GFP cells alone. (B) % GFP fluorescence of CEM-GFP cells + HIV-1_LAI_. (C) % GFP fluorescence of CEM-GFP cells + HIV-1_LAI_ + TFV. (D) % GFP fluorescence of CEM-GFP cells + HIV-1_LAI_ + TFV + *L*. *crispatus*. (E) % GFP fluorescence of CEM-GFP cells + HIV-1_LAI_ + TFV + *G*. *vaginalis*.
(TIF)

S3 Fig Bacterial relative abundance ranked by nugent score. (A) Relative abundance of CVLs from 44 women with and without diagnosed BV grouped by Nugent score. Nugent score cutoffs were 0–3, 4–6, and 7–10. The 19 most abundant phyla are shown. Shannon diversity plots showing alpha diversity in CVLs. Blue, BV negative by Nugent score at the time of collection; Red, BV positive by Nugent score at the time of collection.
(TIF)

S1 Table BV status, HIV status, and microbial community breakdown of WIHS patients. (XLSX)

S2 Table Mathematical modeling of ART drug kinetics reveals that each drug has varying rates of internalization in *G*. *vaginalis* and into target cells. Parameterized values of TAF, TFV and DPV internalization rates into (A) *G*. *vaginalis* and (B) Target (Jurkat) cells and (C) The ratio of internalization rate into Jurkat cell to *G*. *vaginalis*. (* denotes adjusted p < 0.05).
(XLSX)
