# Supplementary figures and images for: Impact of vaginal microbiome communities on HIV antiretroviral-based pre-exposure prophylaxis (PrEP) drug metabolism
Source: PLoS Pathog. 2020 Dec 3;16(12):e1009024. doi: 10.1371/journal.ppat.1009024 (PMC7714160; doi:10.1371/journal.ppat.1009024)

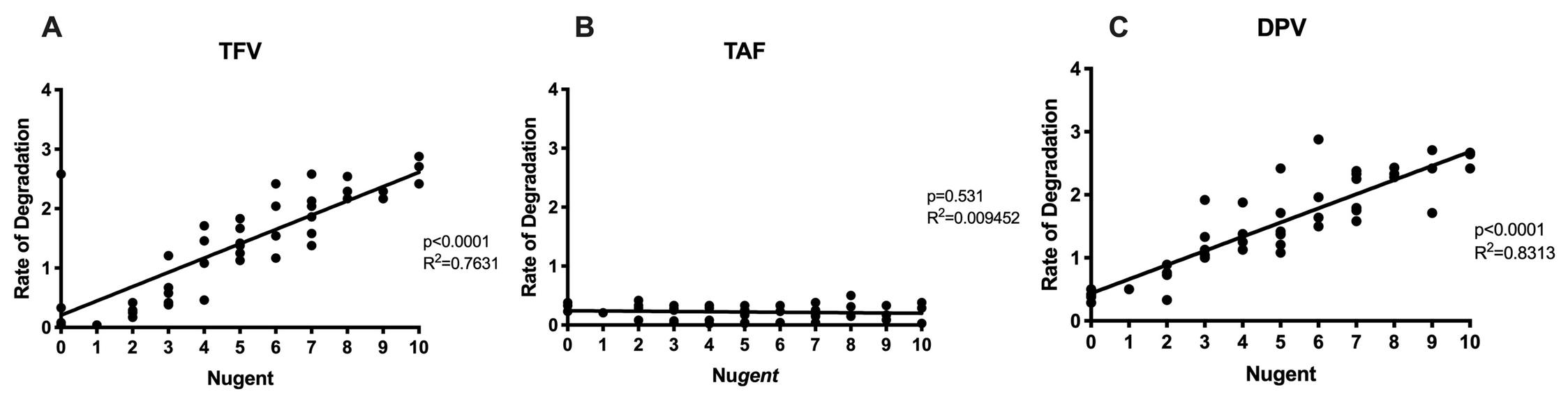

Supplement: S1 Fig — (A) Rate of TFV degradation vs. Nugent score in CVLs after 24-hour incubation with DPV. Rate of degradation calculated as amount lost/24hours. (B) Rate of TAF degradation vs. Nugent score in CVLs after 24-hour incubation with DPV. Rate of degradation calculated as amount lost/24hours. (C) Rate of DPV degradation vs. Nugent score in CVLs after 24-hour incubation with DPV. Rate of degradation calculated as amount lost/24hours. (TIF) [file ppat.1009024.s001.tif]

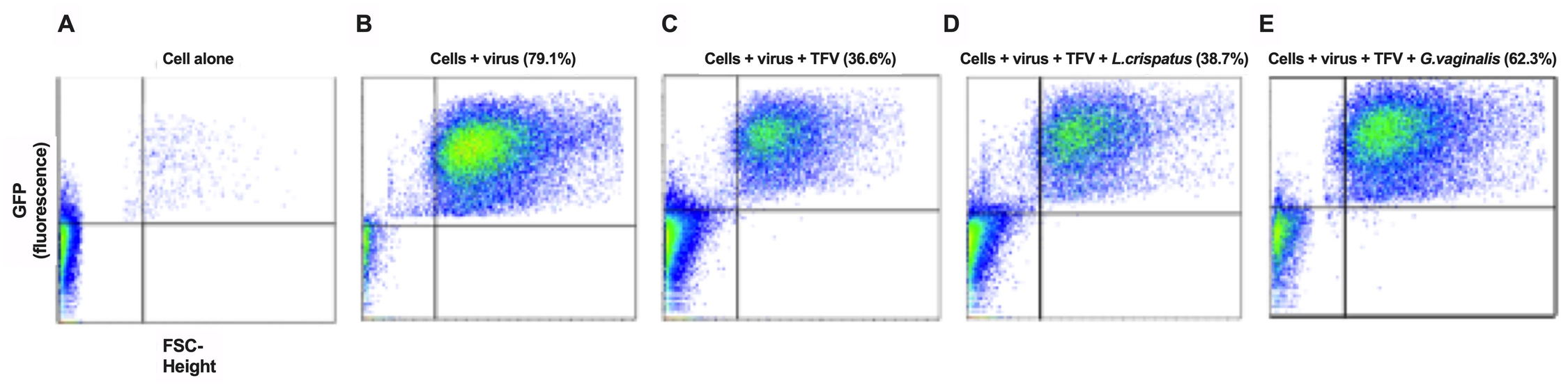

Supplement: S2 Fig — (A) % GFP fluorescence of CEM-GFP cells alone. (B) % GFP fluorescence of CEM-GFP cells + HIV-1LAI. (C) % GFP fluorescence of CEM-GFP cells + HIV-1LAI + TFV. (D) % GFP fluorescence of CEM-GFP cells + HIV-1LAI + TFV + L. crispatus. (E) % GFP fluorescence of CEM-GFP cells + HIV-1LAI + TFV + G. vaginalis. (TIF) [file ppat.1009024.s002.tif]

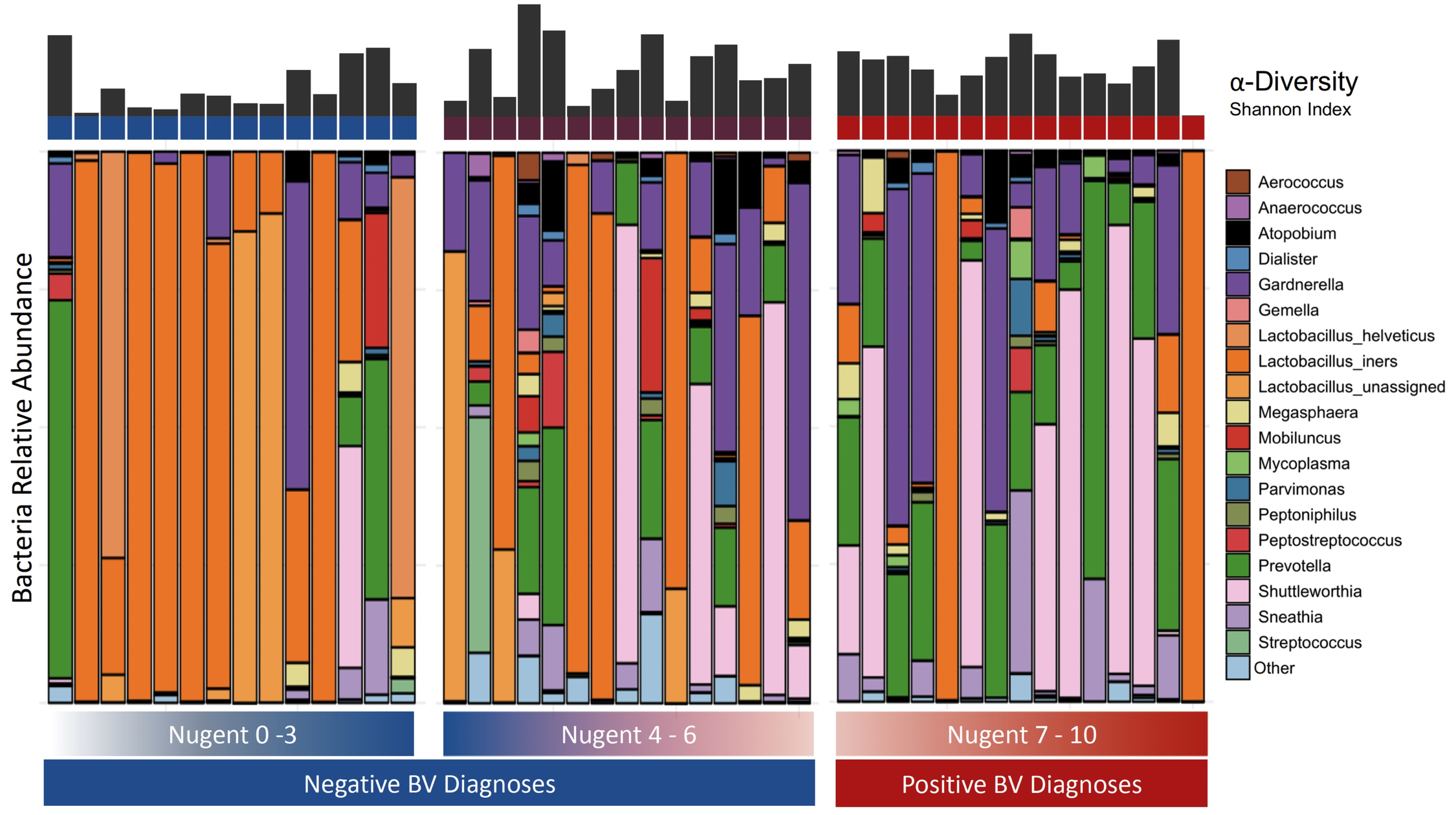

Supplement: S3 Fig — (A) Relative abundance of CVLs from 44 women with and without diagnosed BV grouped by Nugent score. Nugent score cutoffs were 0–3, 4–6, and 7–10. The 19 most abundant phyla are shown. Shannon diversity plots showing alpha diversity in CVLs. Blue, BV negative by Nugent score at the time of collection; Red, BV positive by Nugent score at the time of collection. (TIF) [file ppat.1009024.s003.tif]
